# A minimal markerset for three-dimensional foot function assessment: measuring navicular drop and drift under dynamic conditions

**DOI:** 10.1186/s13047-018-0257-2

**Published:** 2018-04-18

**Authors:** Patric Eichelberger, Angela Blasimann, Nicole Lutz, Fabian Krause, Heiner Baur

**Affiliations:** 1 Bern University of Applied Sciences, Department of Health Professions, Discipline of Physiotherapy, Murtenstrasse 10, Bern, 3008 Switzerland; 2 Graduate School for Cellular and Biomedical Sciences, University of Bern, Bern, Switzerland; 3 University Hospital Bern, Inselspital, Department of Orthopaedic Surgery, Bern, Switzerland

**Keywords:** Navicular mobility, 3D gait analysis, Walking, Instrumental errors

## Abstract

**Background:**

The validity of predicting foot pronation occurring mainly at the midfoot by surrogate measures from the rearfoot, like eversion excursion, is limited. The dynamic navicular mobility in terms of vertical navicular drop (*dNDrop*) and medial navicular drift (*dNDrift*) may be regarded as meaningful clinical indicators to represent overall foot function. This study aimed to develop a minimal approach to measure the two parameters and to examine their intra- and interday reliability during walking.

**Methods:**

The minimal markerset uses markers at the lateral and medial caput of the 1^st^ and 5^th^ metatarsals, respectively, at the dorsal calcaneus and at the tuberosity of the navicular bone. Dynamic navicular drop and drift were assessed with three-dimensional motion capture in 21 healthy individuals using a single-examiner test-retest study design.

**Results:**

Intra- and interday repeatability were 1.1 mm (*ICC*_21_ 0.97) and 2.3 mm (*ICC*_21_ 0.87) for dynamic navicular drop and 1.5 mm (*ICC*_21_ 0.96) and 5.3 mm (*ICC*_21_ 0.46) for dynamic navicular drift. The contribution of instrumental errors was estimated to 0.25 mm for dynamic navicular drop and 0.86 mm for dynamic navicular drift.

**Conclusions:**

Interday reliability was generally worse than intraday reliability primary due to day-to-day variations in movement patterns and the contribution of instrumental errors was below 23% for dynamic navicular drop but reached 57% for dynamic navicular drift. The minimal markerset allows to simply transfer the known concepts of navicular drop and drift from quasi-static clinical test conditions to functional tasks, which is recommended to more closely relate assessments to the functional behavior of the foot.

**Electronic supplementary material:**

The online version of this article (10.1186/s13047-018-0257-2) contains supplementary material, which is available to authorized users.

## Background

Consisting of numerous bones, joints and ligaments, the foot is one of the mechanically more complex structures in the human body [1]. As it is the link to the supporting surface, it is subjected to high impact forces and moments, and proper function is crucial for locomotion since the foot influences the whole kinematic chain [2]. The concept, classifying feet into planus, recuts and cavus based on morphological criteria [1], is still common in foot function evaluation. It is based on the common understanding of the ’excessively pronated’ foot being dysfunctional and the degree of pronation being classified based on structural measures like malleolar valgus index, arch height index or arch height flexibility [1]. Although foot problems, lower leg injury and lower extremity kinematics are often attributed to foot type, causality is not conclusive [3–7]. This is maybe because there is no clear consensus as to whether prediction of dynamic foot function based on structural criteria is reasonable [1, 5, 8], and that clinical measurement of foot pronation is inherently difficult owing to the complex interaction of the joints of the foot when this movement occurs [6, 9]. Furthermore, current methodological limitations in relation to measurement of dynamic foot pronation [5] may be another reason. Conventional measures, like maximum and minimum rearfoot eversion or eversion excursion, are thought to represent foot pronation. These parameters acquired at the rearfoot are likely to be limited for predicting a motion that mainly occurs at the midfoot. It was proposed to merge data on structure with information on foot function under dynamic loading to more closely relate to the functional behaviour of the foot during locomotion [8]. Although numerous foot models to study multi-segment foot kinematics are currently available [10] they do not come up with the requirements of clinical assessments such as being simple and efficient [11] owing to complexity [12] and the knowledge about their reliability [10] is limited.

The vertical navicular drop measures the difference in navicular height between a subtalar neutral and a weight-bearing position [13] and serves as a quick clinical tool for the assessment of a surrogate parameter of midfoot mobility. Similarly, the medial navicular drift was proposed to provide further insight into the mechanics of the talonavicular joint [6]. Both, vertical and mediolateral movements, should be assessed to understand the movement of the midfoot [11] and may be regarded as meaningful clinical indicators to represent overall foot function [6]. Measuring navicular mobility under dynamic conditions by the dynamic navicular drop and drift (*dNDrop*, *dNDrift*) seems to be a promising and intuitive approach to characterise foot function. Evidence shows that the navicular drop [13] is a poor predictor of the dynamic navicular drop [9, 14–16] and that it is therefore necessary to measure the navicular drop dynamically in order to be representative for foot function. Studies that addressed the dynamic navicular drop used either two-dimensional (2D) video technique [14, 16–20], single- or biplane X-ray [9, 21], three-dimensional (3D) motion capture [15] or a stretch sensor device [22, 23]. Although important to describe navicular mobility comprehensively, the dynamic navicular drift has only been examined by Cornwall and McPoil [24]. While some of the 2D studies concerned repeatability [18, 19], the only study that used 3D methods [15] did not consider any reliability issues and did not make use of the potential to measure dynamic navicular drift simultaneously to dynamic navicular drop. The authors recently presented a concept [25] to provide a practical tool to measure clinically accepted foot parameters under dynamic conditions. This study aimed to evaluate the intra- and interday reliability of measuring dynamic navicular drop and drift and the associated time points using the novel concept [25] during the stance phase of walking in healthy adults.

## Methods

### Participants and experimental procedure

The investigation was implemented as a descriptive, cross-sectional laboratory study analysing the intra- and interday reliability of dynamic navicular drop and drift measurements during walking gait. The study was approved by the ethics committee of the canton of Bern (KEK number Z007/12) and all participants signed informed consent. The examined sample consisted of twenty-one healthy participants, 12 females (foot length 239 (SD 11) mm, age 27.9 (SD 5.0) years, height 166 (SD 6) cm, body mass 62.3 (SD 6.9) kg) and nine males (foot length 266 (SD 12) mm, age 32.8 (SD 10.3) years, height 181 (SD 7) cm, body mass 75 (SD 7.4) kg). Exclusion criteria were back pain or other spinal disorders, any kind of musculoskeletal affections, neurological injuries or diseases, thrombosis or fractures in the past 12 months, implants or surgery on the lower extremity in the past 12 months, bone tumours, angiopathies, alcohol abuse, dementia, acute infections (such as common cold) or high-intensity exercise the day before the measurements.

**Fig. 1:**
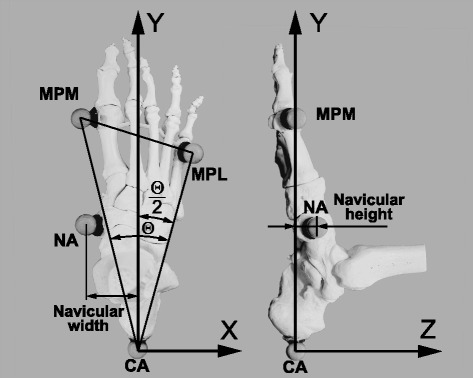
Minimal markerset. Anatomical landmarks with attached markers (NA, MPM, MPL, CA), foot coordinate system and outcome measures navicular height (*NH*) and width (*NW*)

Each subject underwent a test-retest study work flow, where the navicular height (*NH*) and width (*NW*) during the stance phase (SP) were assessed with a set of four markers (Fig. 1) on the left and right foot. A testing session consisted of ten barefoot walking trials and all participants completed two subsequent (no pause between) sessions on a first day (M1a, M1b) to assess intraday reliabiltiy and a third session (M2) one week apart from their first day to assess interday reliability. An optical motion capture system (cameras: 2× Bonita10, 8× Bonita3; volume: 5.5 m × 1.2 m × 2 m; 200 Hz; Nexus 1.8.5 software, Vicon Motion Systems Ltd., Oxford, UK) was used for kinematic measurements. Initial contact and toe off events were determined based on ground reaction force measurements with two force plates (AMTI OR 6, Watertown, USA, 1000 Hz, threshold 25 N).

### Markerset and model outputs

Reflective skin surface markers (diameter 16 mm) were placed by a single experienced physical therapist on the left and the right foot to track the following anatomical landmarks (Fig. 1):

MPM first **M**etatarso-**P**halangeal joint, metatarsal head, dorso-**M**edial aspect.
MPL fifth **M**etatarso-**P**halangeal joint, metatarsal head, dorso-**L**ateral aspect.
CA **CA**lcaneal tuberosity, posterior aspect of the calcaneus (Achilles’ tendon insertion).
NA **NA**vicular tuberosity, most medial aspect of the navicular bone.

MPM, MPL and CA markers served to define the XY-plane (Fig. 1) which was calibrated to be parallel to the foot’s plantar surface based on a static trial and CA marked the origin of a Cartesian coordinate system. The foot’s longitudinal axis (Y) was the bisecting line [26] of the MPM, MPL and CA triangle (Fig. 1). According to the right-hand rule convention, the foot’s vertical axis (Z) was perpendicular to the XY-plane pointing towards cranial and the foot’s lateral axis (X) pointed laterally for the right foot and medially for the left foot. The navicular height described the distance of the navicular marker from the XY-plane and the navicular width described the medial distance of the navicular maker from the YZ-plane.

### Post-processing and statistics

Post-processing and statistical analysis were performed using Matlab (Version R2016a, The MathWorks Inc., Natick, USA). Navicular height and width were calculated and low-pass filtered (4th order zero-lag Butterworth, 4 Hz). Stance phase intervals were extracted, resampled to 200 samples and subsequently averaged among ten trials. Navicular height and width from the static trials (*NH*_S_ and *NW*_S_) were taken as reference positions to express the navicular height and width from gait trials. The curve features, dynamic navicular drop and drift, were extracted from the navicular height and width time-series, respectively, and calculated as differences between minimum (for navicular height, Eq. 1) or maximum (for navicular width, Eq. 2) excursions during the stance phase and values at foot strike (*NH*_FS_, *NW*_FS_):

1 $$\begin{array}{*{20}l} dNDrop & = NH_{\text{FS}} - NH_{\text{Min}}  \end{array} $$

2 $$\begin{array}{*{20}l} dNDrift & = NW_{\text{FS}} - NW_{\text{Max}}  \end{array} $$

Respective time points of dynamic navicular drop and drift were determined in %SP and denoted *tdNDrop* and *tdNDrift*. Reliability was primary analyzed by the Bland-Altman method [27] and completed by the intraclass correlation coefficient *ICC*_21_ and the standard error of measurement (*SEM*, Eq. 3) [28] with *SDd* being the standard deviation of the differences from the Bland-Altman analysis.

3 $$ SEM = \frac{SDd}{\sqrt{2}}   $$

Left and right feet measurements were not treated separately but treated as independent samples making up a total of 42 feet that were assessed at the time points M1a, M1b and M2. Reliability of *NH*_FS_*NH*_Min_*NW*_FS_*NW*_Max_*tdNDrift* and *tdNDrop* was also considered to explore how each parameter contributed to intra- and interday variations in dynamic navicular drop and drift, respectively. All variables and the Bland-Altman differences were tested for normal distribution by the Kolmogorow-Smirnow test. The significance of the bias from the Bland-Altman analysis was evaluated by paired t-tests. The level of statistical significance was set as *p*<0.05.

### Instrumental error assessment

To estimate the instrumental errors, the marker set was applied to a ski boot, which served as a rigid frame. A person wearing the ski boot completed ten walking trials. Model outputs were calculated as described in the “Post-processing” section based on marker distances measured with a caliper. Under the assumption that the markers built a rigid cluster, all marker distances would have been constant throughout the stance phase and deviations from the rigid cluster model outputs were taken for instrumental error estimation by calculating the root mean square errors among the stance phase.

## Results

**Fig. 2:**
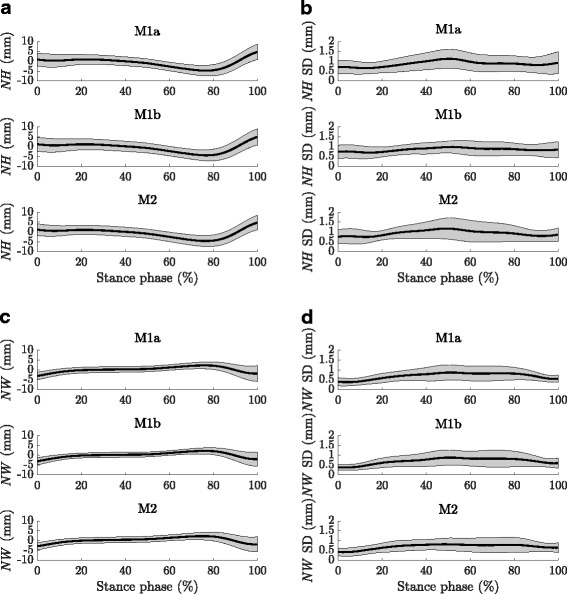
Navicular height and width. Navicular height and width during stance (**a**, **c**) and associated trial-to-trial errors in terms of the standard deviation of ten walking trials (**b**, **d**) averaged among 21 individuals at all three measurement time points. Solid black lines represent the mean, shaded areas the standard deviation. Model outputs from single individuals can be found in Additional file 3. **b** Navicular height trial-to-trial errors. 0.87 mm (M1a), 0.85 mm (M1b) and 0.94 mm (M2) averaged among the stance phase. 0.88 mm overall (M1a to M2). **d** Navicular width trial-to-trial errors. 0.69 mm (M1a), 0.70 mm (M1b) and 0.70 mm (M2) averaged among the stance phase. 0.70 mm overall (M1a to M2)

**Fig. 3:**
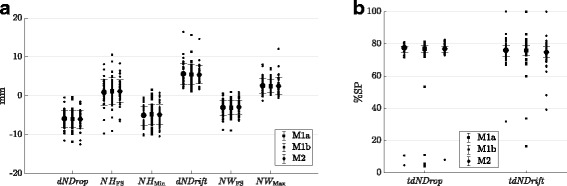
Features extracted from navicular height and width model outputs. Values from 21 individuals with mean ± 1 standard deviation (**a**) and median and 0.25 and 0.75 percentiles (**b**). For the numerical values of the descriptive statistics see Tables 1, 2, 3 and 4

At all three testing sessions the proposed markerset delivered distinct movement patterns of navicular height and width throughout the stance phase among the 21 healthy individuals examined in the present study (Fig. 2a and c). Similar to the mean navicular height and width among all individuals, the standard deviations of 10 walking trials from each subject were averaged among all participants to describe the trial-to-trial errors for each testing session (Fig. 2b and d). Step-to-step variations were relatively constant throughout the stance phase and were between 0.65 and 1.17 mm for navicular height and between 0.38 and 0.88 mm for navicular width. Both, navicular height and width, showed pronounced minima and maxima, respectively, between 74.87 and 77.39% SP (Figs. 2a and c, 3b). A slow caudal drop and medial drift up to around 76% SP followed by a more rapid cranial rise and lateral shift until toe off could have been observed. Except the time point of navicular drop and drift at session M1b, all variables extracted from navicular height and width time-series followed a normal distribution. Complete numerical values of the descriptive statistics are given in the Tables 1, 2, 3 and 4. Related to the static pose, the mean navicular height at foot strike was between 0.9 and 1.2 mm (SD 3.0 to 3.5 mm) and the mean minimum navicular height during the stance phase between -4.8 and -5.1 mm (SD 2.5 to 2.7 mm) (Fig. 3). The resulting dynamic navicular drop was between -5.9 and -6 mm (SD 2.3 mm) (Fig. 3). The mean navicular width at foot strike was between -2.9 and -3.1 mm (SD 1.8 to 1.9 mm) and the mean maximum navicular width during the stance phase was 2.5 mm (SD 1.7 to 2.3 mm) (Fig. 3). The resulting dynamic navicular drift was between 5.4 and 5.6 mm (SD 2.3 to 2.7 mm) (Fig. 3).

**Table 1:** Dynamic navicular drop and associated time points during stance

	*dNDrop*_M1a_	*dNDrop*_M1b_	*dNDrop*_M2_	*tdNDrop*_M1a_	*tdNDrop*_M1b_	*tdNDrop*_M2_
	(mm)	(mm)	(mm)	(%SP)	(%SP)	(%SP)
Mean	− 5.94	− 5.96	− 6.00	73.50	71.60	75.33
sd	2.25	2.25	2.33	15.17	18.72	10.93
Median	− 5.63	− 5.54	− 5.84	77.39	76.88	76.88
Min	− 11.59	− 11.82	− 12.48	4.52	4.02	8.04
Max	− 0.48	− 0.38	− 1.51	80.90	81.41	82.41
KSp	0.949	0.759	0.897	< 0.001	< 0.001	< 0.001

KSp denotes the *p*-value from the Kolmogorow-Smirnow normality distribution test

**Table 2:** Dynamic navicular drift and associated time points during stance

	*dNDrift*_M1a_	*dNDrift*_M1b_	*dNDrift*_M2_	*tdNDrift*_M1a_	*tdNDrift*_M1b_	*tdNDrift*_M2_
	(mm)	(mm)	(mm)	(%SP)	(%SP)	(%SP)
Mean	5.60	5.52	5.41	75.53	74.24	74.02
sd	2.74	2.63	2.29	9.72	13.35	9.15
Median	5.17	5.15	5.25	75.88	75.88	74.87
Min	1.88	1.12	1.69	31.66	16.58	39.20
Max	16.40	15.67	14.62	100.00	100.00	100.00
KSp	0.662	0.231	0.808	0.102	0.019	0.126

KSp denotes the *p*-value from the Kolmogorow-Smirnow normality distribution test

**Table 3:** Navicular height and width at foot strike

	^*NH*^FS_M1a_	^*NH*^FS_M1b_	^*NH*^FS_M2_	^*NW*^FS_M1a_	^*NW*^FS_M1b_	^*NW*^FS_M2_
	(mm)	(mm)	(mm)	(mm)	(mm)	(mm)
Mean	0.86	1.20	1.15	− 3.05	− 3.06	− 2.88
sd	3.27	3.53	2.98	1.92	1.80	1.82
Median	1.32	1.39	1.47	− 2.79	− 3.22	− 2.76
Min	− 9.76	− 9.05	− 6.49	− 8.83	− 8.91	− 6.40
Max	8.08	10.57	8.34	0.58	1.00	0.94
KSp	0.826	0.760	0.901	0.910	0.830	0.966

KSp denotes the *p*-value from the Kolmogorow-Smirnow normality distribution test

**Table 4:** Minimum and maximum navicular height and width during stance

	^*NH*^Min_M1a_	^*NH*^Min_M1b_	^*NH*^Min_M2_	^*NW*^Max_M1a_	^*NW*^Max_M1b_	^*NW*^Max_M2_
	(mm)	(mm)	(mm)	(mm)	(mm)	(mm)
Mean	− 5.08	− 4.76	− 4.85	2.54	2.46	2.52
sd	2.46	2.67	2.62	1.89	1.72	2.29
Median	− 4.85	− 4.48	− 4.46	2.49	2.35	2.22
Min	− 10.24	− 9.97	− 10.46	− 1.33	0.20	− 0.55
Max	− 0.53	1.54	0.82	8.01	7.13	12.08
KSp	0.901	0.808	0.744	0.331	0.533	0.203

KSp denotes the *p*-value from the Kolmogorow-Smirnow normality distribution test

### Reliability

Since the article focuses on measuring navicular drop and drift, Bland-Altman diagrams are presented for these outcomes only in the main part of the manuscript. For the Bland-Altman diagrams of the other variables the reader is referred to the Additional file 1. None of the considered variables had a significant intra- or interday bias.

**Fig. 4:**
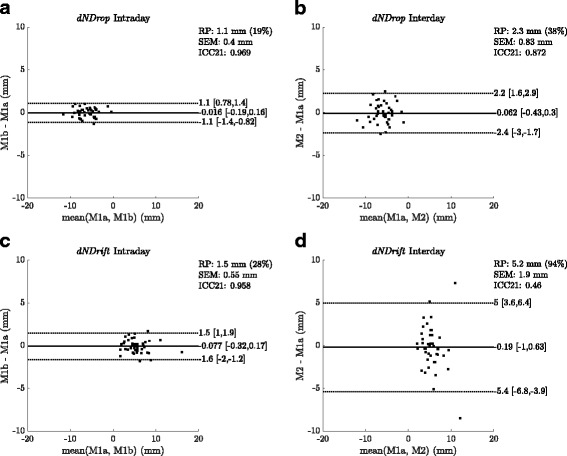
Reliabiltiy of *dNDrop* and *dNDrift*. Bland-Altman diagrams with *SEM* and *ICC*_21_ for intra- and interday reliability of *dNDrop* (**a**) (**b**) and *dNDrift* (**c**) (**d**). *RP* denotes the repeatability calculated as 1.96 x *SDd* (standard deviation of the differences) which is also given as a percentage of the mean dynamic navicular drop and drift, respectively. Solid black lines: bias; Dotted lines: limits of agreement (LoA) calculated as bias ±*RP*; Numbers in brackets: 95% confidence intervals of bias and LoAs, respectively

The intra- and interday repeatability of the dynamic navicular drop were found to be 1.1 and 2.3 mm, those of the dynamic navicular drift 1.5 and 5.2 mm (Fig. 4), respectively. Associated intraclass correlation coefficients were found to be 0.97 and 0.83 for the dynamic navicular drop and 0.96 and 0.46 for the navicular drift (Fig. 4).

Intra- and interday repeatability of navicular height at foot strike were 2.7 mm (*ICC*_21_ 0.91) and 4.9 mm (*ICC*_21_ 0.68) and those of the minimum navicular height were 2.8 mm (*ICC*_21_ 0.85) and 4.7 mm (*ICC*_21_ 0.55). The intra- and interday repeatability of the navicular width at foot strike were 2.3 mm (*ICC*_21_ 0.81) and 3.7 mm (*ICC*_21_ 0.49) and those of the maximum navicular width were 2.7 mm (*ICC*_21_ 0.72) and 4.7 mm (*ICC*_21_ 0.35).

The time point of the dynamic navicular drop had an intra- and interday repeatability of 22% SP (*ICC*_21_ 0.79) and 21% SP (*ICC*_21_ 0.67), respectively. The time point of the dynamic navicular drift had an intra- and interday repeatability of 26% SP (*ICC*_21_ 0.36) and 20% SP (*ICC*_21_ 0.43), respectively.

### Instrumental errors

**Fig. 5:**
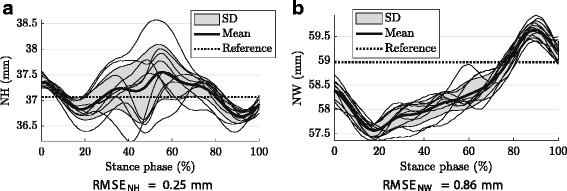
Instrumental errors. Navicular height (**a**) and width (**b**) from instrumental error assessments with a rigid marker cluster. Thin lines: single walking trials; Bold line: mean; Shaded area: standard deviation; Dashed line: rigid marker cluster reference. Errors among the stance phase (*RMSE*_NH_ and *RMSE*_NW_) quantified as root mean square of the deviation of the mean from the reference

The mean deviations from the reference, i.e. the instrumental errors, had some kind of systematic patters over the stance phase, especially the navicular width model output (Fig. 5). The instrumental errors were in minimum and maximum -0.38 mm and 0.48 mm, respectively, for navicular height and -1.40 mm and 0.67 mm, respectively, for navicular width (Fig. 5). Quantified as root mean square errors over the stance phase, the instrumental error was *RMSE*_NH_ = 0.25 mm for the navicular height and *RMSE*_NW_ = 0.86 mm for the navicular width (Fig. 5).

## Discussion

We developed a minimal markerset to dynamically assess foot function by means of navicular mobility and examined the intra- and interday reliability of the dynamic navicular drop and drift as measures for the maximum vertical and medial displacements of the navicular during the stance phase of walking. The investigation revealed navicular height stance phase patterns and dynamic navicular drop values that are consistent with current knowledge. The observed navicular height patterns resembled those previously presented by other authors [15, 22, 23], who also found the navicular height being decreasing after foot strike with a characteristic minimum around 75% SP, followed by a subsequent increase during the remaining stance phase. The mean dynamic navicular drop from this study (6.0 mm) was below those which Dicharry [15] found in hypomobile, neutral and hypermobile feet (7.9 to 8.3 mm) with a similar 3D measurement approach but were slightly higher or similar to those Nielsen et al. [17] measured with a 2D approach in supinated, neutral and pronated feet. Compared to values that were presented as reference data based on 79 healthy participants measured with 2D video (mean 5.4±1.7 mm, range [1.3,9.2] mm), the values from this study were on average slightly higher but exhibited an extended range (0.4 to 12.5 mm). Compared with the only reference for the dynamic navicular drift [24] (4.7±2.0 mm) known to the authors, the values from this study were slightly higher. We found the following stance phase pattern for the navicular: caudal drop and medial drift up to 75% SP followed by subsequent cranial rise and lateral drift. This complies with the common understanding of normal foot function with pronation and arch flattening during loading response and midstance followed by supination and arch rise during push-off to stiffen the foot for effective propulsion power generation [2]. Discrepancies in dynamic navicular drop may be explained by uncontrolled foot lengths which are thought to influence the dynamic navicular drop [18], inclusion of participants without classifying feet and the limited sample size of only 21 participants. Hence the presented data should not be regarded as representative normative data but the outlined measurements can be considered with well provided external validity. Further, as for every skin-surface marker based movement analysis protocol, whether 2D or 3D, soft tissue artifacts (STAs) should always be borne in mind in terms of internal validity. The navicular height has been shown to be overestimated of about 1-2 mm during the first half of stance but up to 12 mm during terminal stance [29]. Hence, the proposed protocol, as well as similar protocols already known from literature, do most likely not exactly measure the position of the navicular tuberosity. However, the displacement patterns seem to be well represented by skin-surface marker based approaches, which may deliver more clinically important information about dynamic foot function compared with static assessments.

For both, the dynamic navicular drop and drift, the interday reliability was worse than the intraday reliability: repeatability and *SEM* were larger and *ICC*_21_ values smaller. The ICC values indicate that the dynamic navicular drop can be reliably measured within and between days but that the dynamic navicular drift is only reliably measurable within day. However the conclusions based on ICCs have to be drawn carefully, because ICCs are dependent from the sample heterogeneity [28], i.e. the actual values of dynamic navicular drop and drift, respectively. The values reported as repeatability are absolute measures of measurement error and quantify the minimal detectable changes for within and between day assessments. Hence, changes in dynamic navicular drop must be in minimum 1.1 mm (19%) and 2.3 mm (38%) to be detectable in within day and between day assessments, respectively. For the dynamic navicular drift, changes must be in minimum 1.5 mm (28%) for within day assessments to be detectable. Reliability of the time points of dynamic navicular drop and drift was negatively influenced by outliers resulting from participants that did not show a clear minimum navicular height or maximum navicular width around 76% SP (see Additional file 1). Disregarding these outliers, the time points of dynamic navicular drop and drift would have been measurable very consistently. All curve parameters from which the dynamic navicular drop and drift were calculated showed poorer reliability than the dynamic navicular drop and drift themselves (repeatability of about factor two increased). Their repeatability was larger for interday measurements but respectively equal for foot strike and minimum/maximum measures. Hence, whether values at foot strike nor minimum or maximum values were predominating the reliability of the dynamic navicular drop and drift and the reliability of the range of motion measures was superior than single features of navicular height and width during the stance phase.

The study was the first that investigated the reliability of 3D approaches to measure dynamic navicular drop and drift and found that both are measurable with similar and good reliability during intraday assessments. For interday assessments, only the dynamic navicular drop can be recommended.

Reliability is influenced by the examiner (marker placement errors), the laboratory setup [30] (instrumental error) and the participants (step-to-step and intersession errors) which together make up the total error in gait analysis based on multiple trials [31]. Instrumental errors, which were previously optimized [30], and step-to-step errors contributed to the reported trial-to-trial errors (Fig. 2). Intraday reliability was influenced by trial-to-trial and intersession errors, interday reliability additionally by potential marker placement errors. The latter are thought to be of minor importance for the current investigation because: (1) a calibration routine guaranteed the reference planes for measuring navicular height and width being properly aligned and (2) the same experienced examiner placed the markers. Hence, the larger interday reliabilities would have been primary induced by increased intersession errors during interday compared with intraday assessments. This may be explained by learning effects or natural day-to-day variations in movement patterns. For example, if participants were initially not familiar with walking barefoot they could have become more aware about how to walk barefoot from participating in the study that potentially affected movement patterns during the repeated assessments. Further, it has to be acknowledged that the experiments were carried out under laboratory conditions, where for example targeting the force plates might have initially bothered participants from walking naturally. Hence, that participants learned to deal with the laboratory conditions with increasing practice might have been another learning effect that affected movement patterns.

## Conclusions

In contrast to recent multi-segment models consisting of many markers, relying on rigid body assumptions and delivering rather complex 3D rotations to characterize foot function, the presented minimal approach uses projections of the trajectory of the navicular tuberosity to the foot’s transverse and sagital planes. This allows to transfer the known concept of navicular drop and drift assessment from quasi-static clinical test conditions to functional tasks, which is recommended to more closely relate assessments to the functional behavior of the foot [8]. We hope that simultaneously measuring medio-lateral in addition to vertical navicular movement will add value to foot function assessment in the future. We propose a concept which allows adding intrinsic foot movement measurements to standard 3D gait analysis protocols. Using a minimal markerset consisting of only four markers provides a “lightweight” approach in terms of preparation time, laboratory infrastructure requirements and post-processing efforts. Validating the proposed measurements of navicular mobility against a multi-segment foot model would be valuable in the future to further substantiate the use of dynamic naviular drop and drift as parameters for foot function. Further, it should be evaluated if the proposed model outputs may discriminate patient groups, reveal intervention effects or identify risk factors for injury and dysfunction.

## Additional files

Additional file 1 Bland-Altman diagrams for navicular height and width at foot strike and minimum and maximum of navicular height and width, respectively, during stance. Bland-Altman diagrams for the time points of minimum navicular height and maximum navicular width are also presented. (PDF 711 kb)

Additional file 2 Dataset. The dataset used for this article. A ZIP-archive with one text file (comma separated values;.csv) per participant, containing the stance-normalized navicular height and width time series from all analyzed steps. (ZIP 8335 kb)

Additional file 3 Individual model outputs. Navicular height and width during stance from all individuals. (PDF 2324 kb)

## Abbreviations

CA: Skin surface marker calcaneus
dNDrift: Dynamic navicular drift
dNDrop: Dynamic navicular drop
FS: Foot strike
ICC: Intraclass correlation coefficient
LoA: Limits of agreement
M1a: First assessment on day 1
M1b: Second assessment on day 1
M2: Assessment on day 2
MPL: Skin surface marker 5th metatarsal head
MPM: Skin surface marker 1st metatarsal head
NA: Skin surface marker navicular tuberosity
NH: Navicular height
NH_FS_: Navicular height at foot strike
NH_Min_: Minimum navicular height during stance
NH_S_: Navicular height during static trial
NW: Navicular width
NW_FS_: Navicular width at foot strike
NW_Max_: Maximum navicular width during stance
NW_S_: Navicular width during static trial
RMSE: Root mean square error
RP: Repeatability
SD: Standard deviation
SDd: Standard deviation of the test-retest differences
SEM: Standard error of measurement
SP: Stance phase
