# Supplementary material for: A minimal markerset for three-dimensional foot function assessment: measuring navicular drop and drift under dynamic conditions
Source: J Foot Ankle Res. 2018 Apr 18;11:15. doi: 10.1186/s13047-018-0257-2 (PMC5907216; doi:10.1186/s13047-018-0257-2)
Supplement: Supplementary file 1 — Bland-Altman diagrams for navicular height and width at foot strike and minimum and maximum of navicular height and width, respectively, during stance. Bland-Altman diagrams for the time points of minimum navicular height and maximum navicular width are also presented. (PDF 711 kb) [file 13047_2018_257_MOESM1_ESM.pdf]

Additional file 1

Contents

1 **Bland-Altman diagrams of navicular height and width at foot strike** 2

2 **Bland-Altman diagrams of minimum navicular height and maximum navicular width** 3

3 **Bland-Altman diagrams from time points of minimum navicular height and maximum navicular width** 4

1 Bland-Altman diagrams of navicular height and width at foot strike

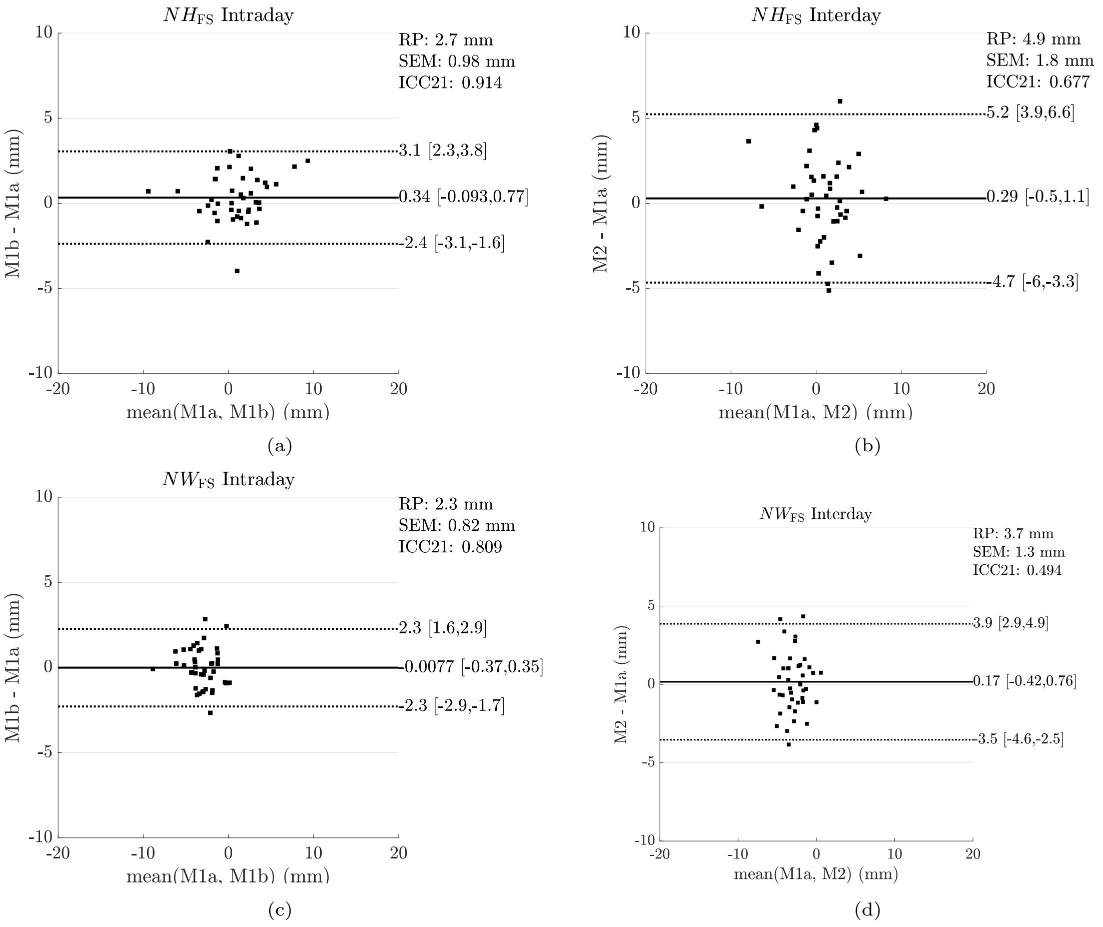

Figure 1: Bland-Altman diagrams with *SEM* and ICC_{21} for intra- and interday reliability of navicular height (NH_{FS}) **(a)** **(b)** and width (NW_{FS}) **(c)** **(d)** at foot strike. *RP* denotes the repeatability calculated as 1.96 x *SDd* (standard deviation of the differences). Solid black lines: bias; Dotted lines: limits of agreement (LoA) calculated as bias ± *RP*; Numbers in brackets: 95% confidence intervals of bias and LoAs, respectively.

2 Bland-Altman diagrams of minimum navicular height and maximum navicular width

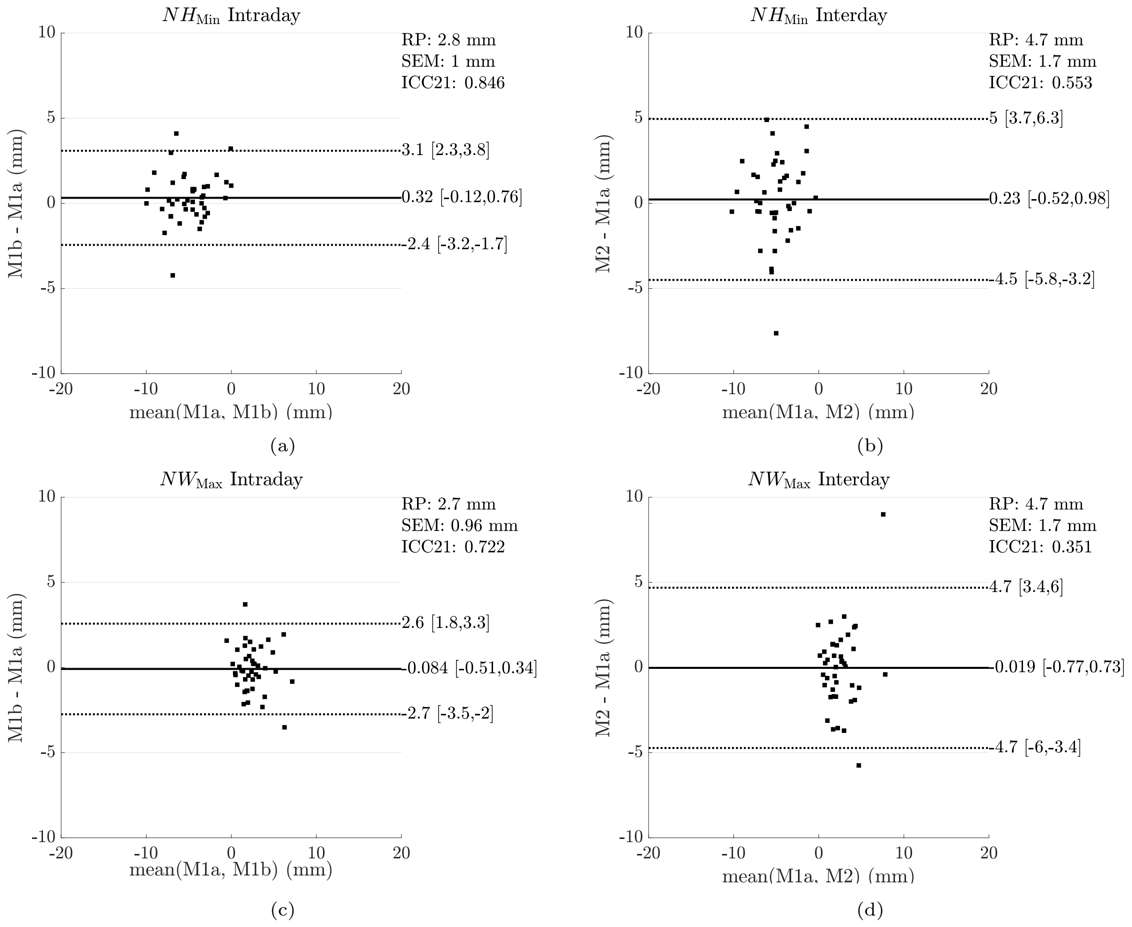

Figure 2: Bland-Altman diagrams with SEM and ICC_{21} for intra- and interday reliability of minimum navicular height (NH_{Min}) **(a)** **(b)** and maximum navicular width (NW_{Max}) **(c)** **(d)** during the stance phase. RP denotes the repeatability calculated as 1.96 x SDd (standard deviation of the differences). Solid black lines: bias; Dotted lines: limits of agreement (LoA) calculated as bias $\pm$ RP; Numbers in brackets: 95% confidence intervals of bias and LoAs, respectively.

3 Bland-Altman diagrams from time points of minimum navicular height and maximum navicular width

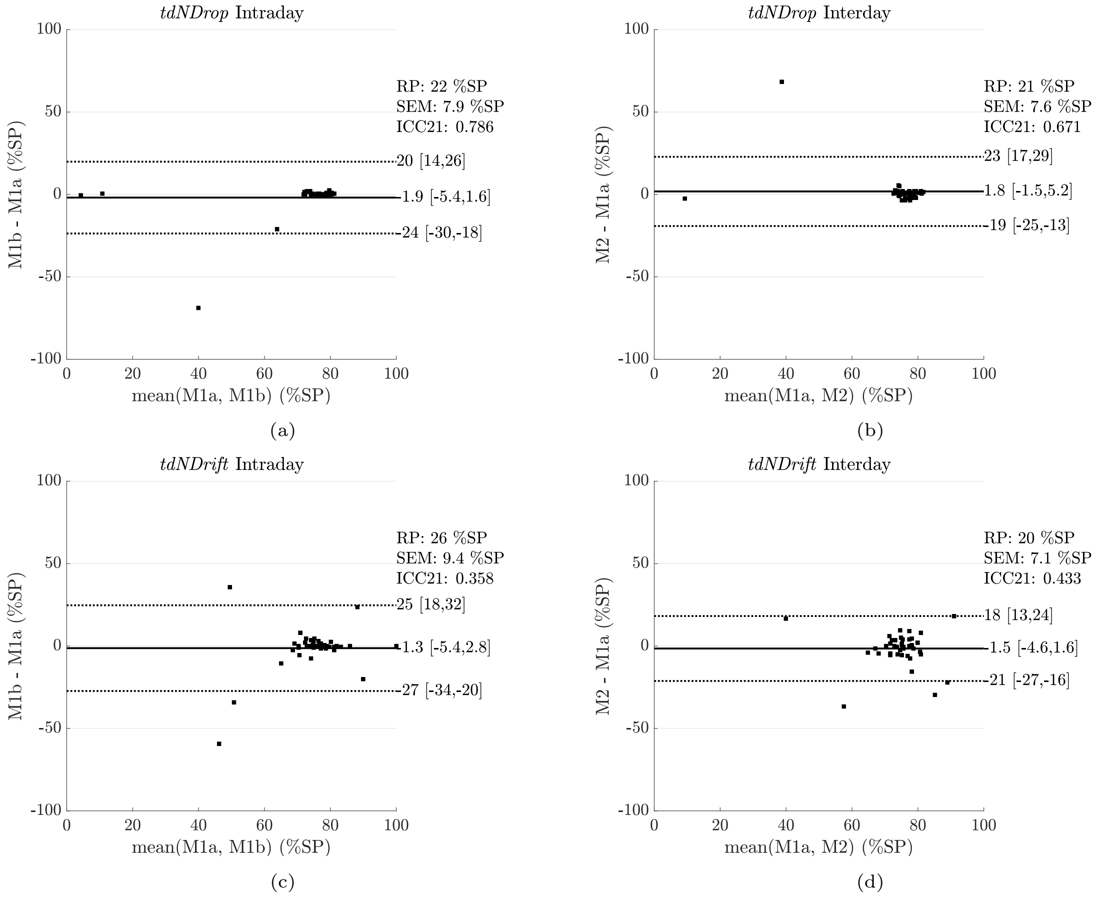

Figure 3: Bland-Altman diagrams with SEM and ICC_{21} for intra- and interday reliability of the time points of dynamic navicular drop (*tdNDrop*) **(a)** **(b)** and drift (*tdNDrift*) **(c)** **(d)** during the stance phase. RP denotes the repeatability calculated as 1.96 x SDd (standard deviation of the differences). Solid black lines: bias; Dotted lines: limits of agreement (LoA) calculated as bias $\pm$ RP; Numbers in brackets: 95% confidence intervals of bias and LoAs, respectively.
