# Supplementary figures and images for: A minimal markerset for three-dimensional foot function assessment: measuring navicular drop and drift under dynamic conditions
Source: J Foot Ankle Res. 2018 Apr 18;11:15. doi: 10.1186/s13047-018-0257-2 (PMC5907216; doi:10.1186/s13047-018-0257-2)

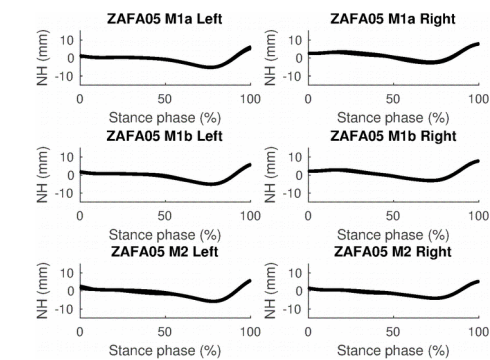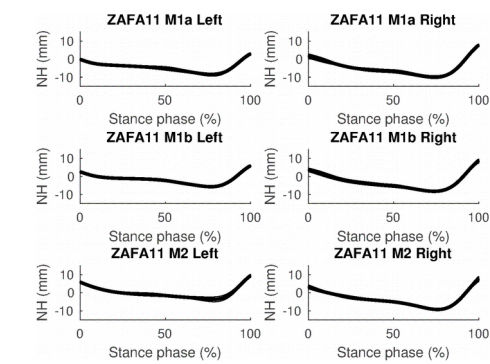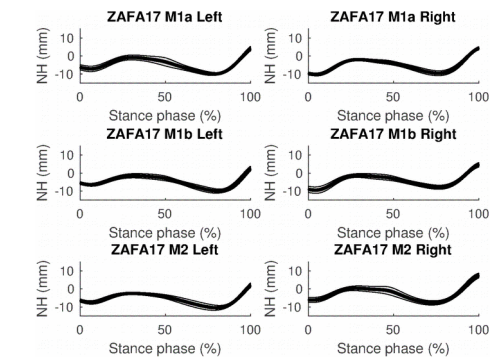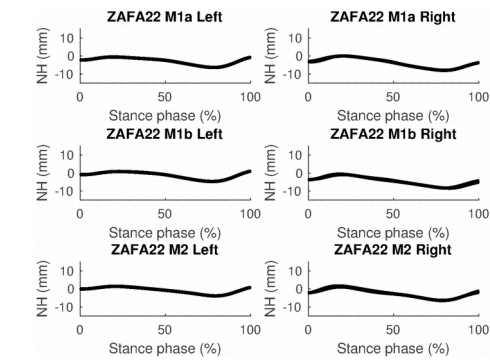

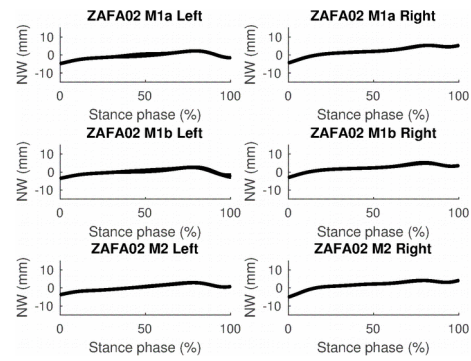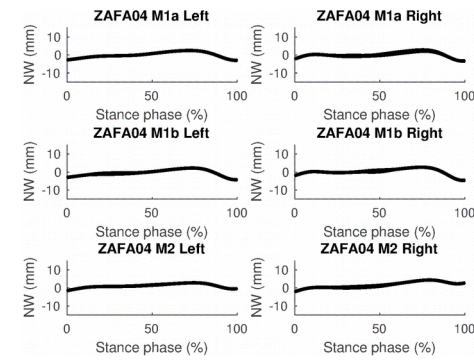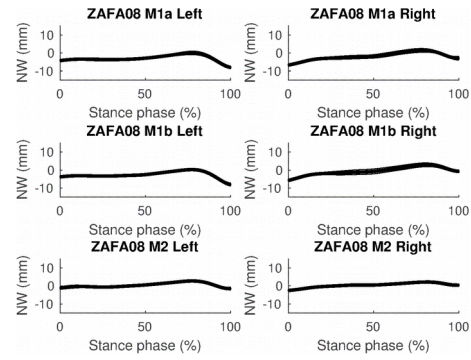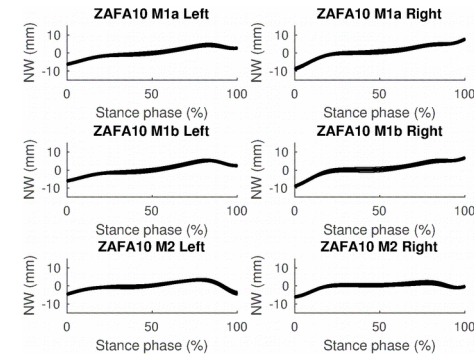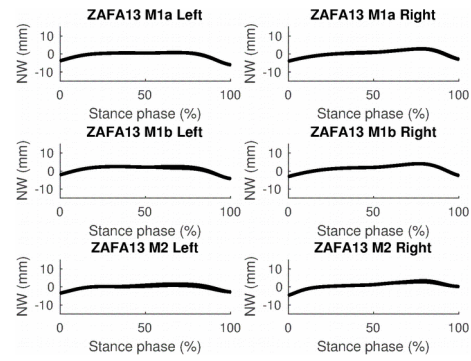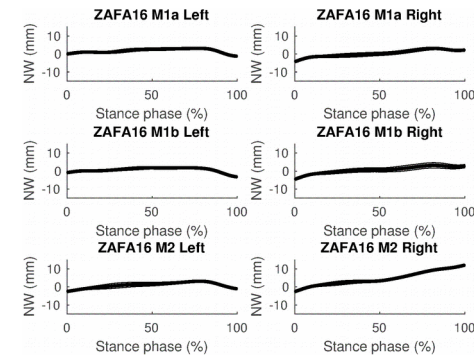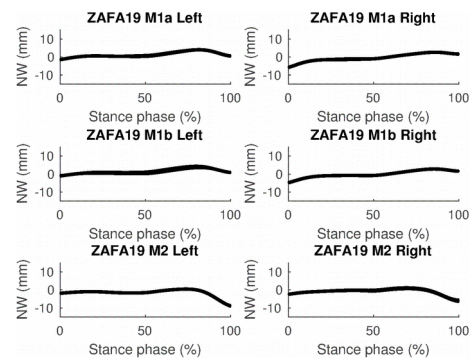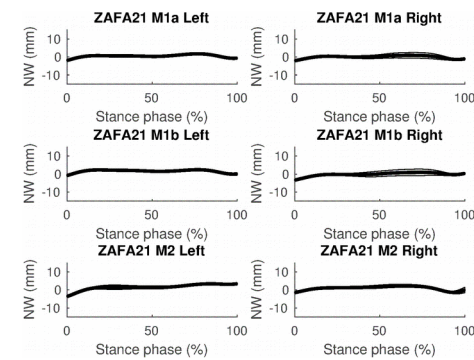

Supplement: Supplementary file 3 — Individual model outputs. Navicular height and width during stance from all individuals. (PDF 2324 kb) [file 13047_2018_257_MOESM3_ESM.pdf]
